# Neurotrophin Promotes Neurite Outgrowth by Inhibiting Rif GTPase Activation Downstream of MAPKs and PI3K Signaling

**DOI:** 10.3390/ijms18010148

**Published:** 2017-01-13

**Authors:** Xiaoxia Tian, Huijuan Yan, Jiayi Li, Shuang Wu, Junyu Wang, Lifei Fan

**Affiliations:** School of Life Sciences, Inner Mongolia University, Hohhot 010021, China; xiaoxiatian912@163.com (X.T.); huhehaoteyhj@163.com (H.Y.); MandyLjy1996@163.com (J.L.); flieish0376@163.com (S.W.); 13074782860@163.com (J.W.)

**Keywords:** semaphorin 6A, neurotrophin, neurite outgrowth, Rif GTPase

## Abstract

Members of the well-known semaphorin family of proteins can induce both repulsive and attractive signaling in neural network formation and their cytoskeletal effects are mediated in part by small guanosine 5’-triphosphatase (GTPases). The aim of this study was to investigate the cellular role of Rif GTPase in the neurotrophin-induced neurite outgrowth. By using PC12 cells which are known to cease dividing and begin to show neurite outgrowth responding to nerve growth factor (NGF), we found that semaphorin 6A was as effective as nerve growth factor at stimulating neurite outgrowth in PC12 cells, and that its neurotrophic effect was transmitted through signaling by mitogen-activated protein kinases (MAPKs) and phosphatidylinositol-3-kinase (PI3K). We further found that neurotrophin-induced neurite formation in PC12 cells could be partially mediated by inhibition of Rif GTPase activity downstream of MAPKs and PI3K signaling. In conclusion, we newly identified Rif as a regulator of the cytoskeletal rearrangement mediated by semaphorins.

## 1. Introduction

Semaphorins were initially identified as axonal repellents in central nervous system development [1]. Over the last few years, semaphorins were shown to have both repulsive and attractive functions, similar to members of most other guidance cue families. Moreover, many semaphorins are bifunctional [2]. The transmembrane protein semaphorin 6A (Sema6A) was initially identified as a repulsive axon guidance cue [3]. It was also revealed to promote the dendritic growth of lateral motor column neurons through the Sema6A/plexinA4/FERM, RhoGEF, and pleckstrin homology domain protein 1(FARP1) signaling pathway [4].

The PC12 cell line is used as a model system to study neuronal differentiation or dissecting the dendrites extension pathways, as they cease dividing and begin to show neurite outgrowth. This process occurs after nerve growth factor (NGF) binds to its tyrosine kinase receptor Trk, leading to neuronal differentiation by the Ras-mitogen activated protein kinase (MAPK) signaling pathway [5]. MAPK family members act as integration points for multiple biochemical signals, and they are also involved in a variety of cellular processes, such as proliferation, differentiation, transcriptional regulation, and development. MAPK is activated through its phosphorylation by upstream kinases [6]. Receptor-linked tyrosine kinases, Ras, Raf, mitogen activated protein kinase kinase 1 (MEK), and MAPK are part of cascades linking extracellular signals to MAPK activation [7]. In addition, several growth factors are regarded as mitogenic stimuli participating in neuron–glia cross-talk, inducing proliferation and differentiation in nerve cell cultures [8,9]. The neurotrophic effects of semaphorins were also investigated, showing that activation of MAPK by Sema3A was crucial for PC12 cell differentiation and that protein kinase C (PKC), l-type voltage-dependent Ca^2+^ channels and PI3K could mediate the neurotrophic actions of Sema4D in PC12 cells [10,11].

Small GTPases play pivotal roles in regulating neuronal morphology and migration, depending on dynamic regulation of the actin cytoskeleton. Three well-studied Rho GTPases, RhoA, Rac1, and Cdc42, were all implicated in neuronal differentiation in PC12 cells. Rho activation caused neurite retraction and cell rounding, whereas Rac and Cdc42 were implicated in neurite outgrowth [12]. Less-studied Rho GTPases, such as Rnd and RhoG, also were shown to regulate neuronal differentiation in PC12 cells. Rnd1 induced the Rac-dependent neuritic process formation [13,14]. Rnd2 regulated neurite outgrowth by functioning as a RhoA activator, in contrast to Rnd1 and Rnd3/RhoE, both of which inhibited RhoA signaling [15,16,17]. RhoG was reported to be a key regulator for neurite outgrowth, working upstream of Rac1 and Cdc42 and downstream of Ras in PC12 cells [18,19,20]. As a newly described addition to the Rho family, Rif was observed only in chordates, displaying a relatively low homology to other family members. Rif exhibited multiple functions in actin cytoskeleton remodeling. It controlled filopodia formation, through diaphanous-related formin mDia2, and acted as an alternative trigger for actin stress fibers formation in epithelial cells, via diaphanous-related formin mDia1 [21,22]. Rif was also shown to play pivotal roles in other physiological processes, such as dentritic spine formation, early-stage mycosis fungoides, and follicular lymphoma development [23,24,25].

Previously, we demonstrated that Rif participated in the release of active RhoA from FARP1 in a pathway that linked it to regulation of repulsive signaling by Sema6A [26]. The aim of our present study was to investigate the cellular role and signaling pathways of Rif GTPase in neurotrophin promoted neurite outgrowth. We found that, in PC12 cells, neurotrophin promoted neurite outgrowth partially by inhibiting Rif GTPase activation downstream of MAPK and PI3K signaling. Hence, Rif played a pivotal role in RhoA inactivation in PC12 cells. This was an alternative mechanism to that of classical RhoA inactivation pathways involving self-phosphorylation, 190 Rho GTPase activating protein (190RhoGAP), and ArfGAP with RhoGAP domain, ankyrin repeat and PH domain 3 (ARAP3) in response to di-butyric cAMP [27].

## 2. Results

### 2.1. Semaphorin 6A (Sema6A) Was as Effectively Neurotrophic as Nerve Growth Factor (NGF) in PC12 Cells

**Figure 1:**
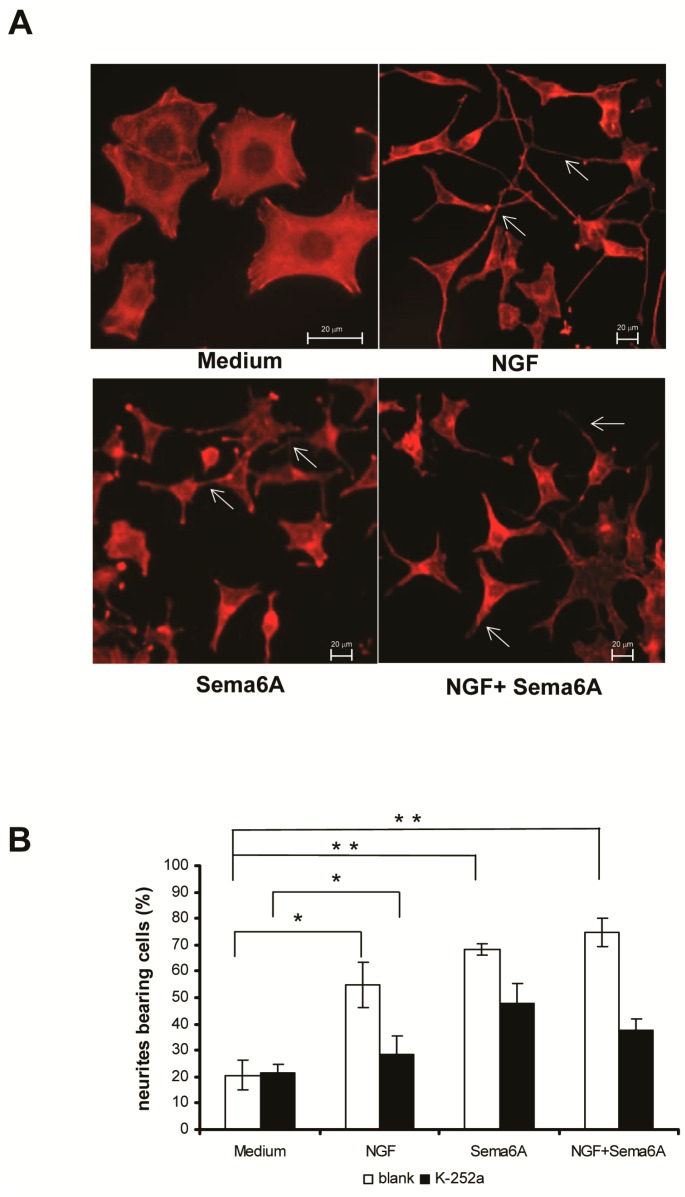
Semaphorin 6A (Sema6A) induced neurite outgrowth in PC12 cells. (**A**) PC12 cells were cultured in RPMI-1640 containing 50 ng/mL NGF and/or 1 nM Sema6A for 24 h and then the cell cytoskeleton stained with Alexa Fluor^®^ 594 phalloidin (ThermoFisher Scientific, Waltham, MA, USA). PC12 cells cultured in normal RPMI-1640 medium were used as controls. Arrows indicate the represented cells with neuritis; (**B**) PC12 cells were pretreated with 100 nM K-252a for 30 min. Next, 50 ng/mL nerve growth factor (NGF) and/or 1 nM Sema6A were added to the medium, cells incubated for another 24 h and the cytoskeleton stained with Alexa Fluor^®^ 594 phalloidin. Neurite outgrowth was quantified by counting PC12 cells bearing at least one neurite with a length longer than twice the cell body diameter. Each value is the mean ± standard error (S.E.) for >100 PC12 cells from three independent experiments. (* *p* < 0.05, ** *p* < 0.01).

It is widely accepted that NGF stimulates neurite outgrowth in PC12 cells and we further found that incubation of PC12 cells with Sema6A for 24 h induced neurite extension similar to that with NGF. On the contrary, little or no morphological differentiation occurred in PC12 cells incubated with control medium (Figure 1A). In PC12 cells treated with the NGF receptor (TrkA) inhibitor K-252a, NGF-stimulated neurite outgrowth was nearly completely blocked, while that induced by Sema6A was partially blocked (Figure 1B). This suggested that a receptor other than a Trk receptor, such as plexinA4, mediated the effects of Sema6A in PC12 cells. In the presence of NGF, Sema6A treatment led to an increased number of PC12 cells possessing neurites, compared with cells treated with NGF alone (Figure 1B). This may have been an indirect effect of Sema6A on neurite outgrowth, increasing the sensitivity of PC12 cells to NGF [11]. These results showed that Sema6A had neurotrophic effects in PC12 cells.

### 2.2. Mitogen-Activated Protein Kinase and Phosphoinositide 3-Kinase Signaling Were Necessary for Sema6A-Stimulated NGF-Induced Neurite Outgrowth in PC12 Cells

**Figure 2:**
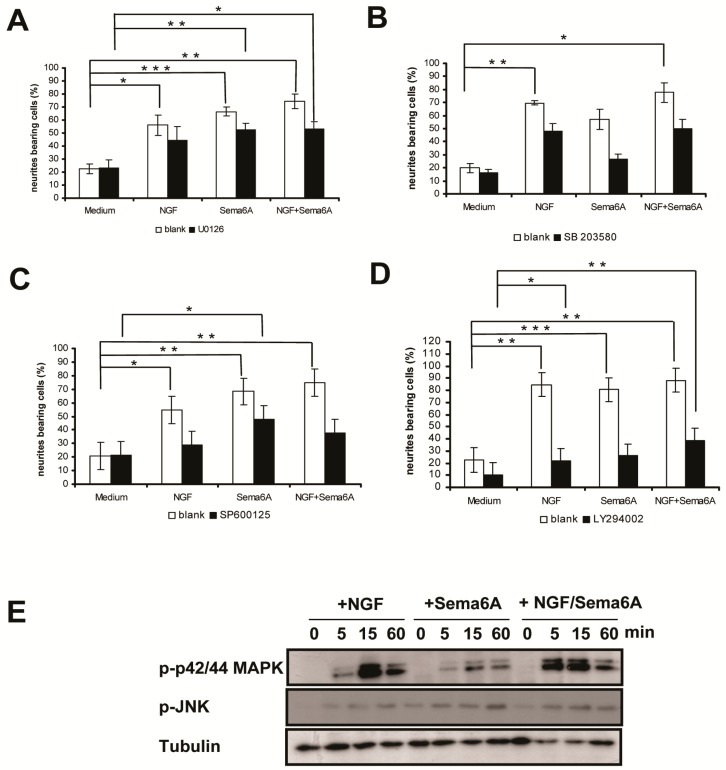
Mitogen-activated protein kinases (MAPKs) and phosphatidylinositol-3-kinase (PI3K) signaling were necessary for Sema6A-stimulated NGF-induced neurite outgrowth in PC12 cells. PC12 cells were pretreated for 30 min with (black bar) or without (white bar) 10 µm p42/44 MAPK inhibitor U0126 (**A**); 10 µm p38 MAPK inhibitor SB203580 (**B**); 50 µm c-jun NH_2_-terminal kinase (JNK) MAPK inhibitor SP600125 (**C**) or 50 µm PI3K inhibitor LY294002 (**D**) prior to 50 ng/mL NGF and/or 1 nM Sema6A. Neurite outgrowth was quantified by counting PC12 cells bearing at least one neurite with a length longer than twice the cell body diameter. Each value is the mean ± S.E. for >100 PC12 cells sampled from three independent experiments. * *p* < 0.05; ** *p* < 0.01; *** *p* < 0.001, respectively, by Student’s *t* test; (**E**) Time course experiment of MAPK phosphorylation in PC12 cells after being treated with 50 ng/mL NGF and/or 1 nM Sema6A. PC12 cells were treated for 5, 15, or 60 min with NGF, Sema6A or NGF + Sema6A. At the indicated times, phospho-p42/p44 MAPK (p-p42/p44 MAPK) or phospho-JNK (p-JNK) were detected by immunoblotting with antibodies recognizing the corresponding phosphorylated proteins.

It was previously shown that an NGF-induced sustained activation of the MAPK pathway was crucial to neuronal differentiation of PC12 cells [28,29]. Other evidence suggested that MAPK activation was essential for neurite outgrowth induced by Sema4D, Sema3A, or mouse semaphorin H, implicating MAPK activation as a common downstream effect of semaphorin signaling [10,11,30]. Therefore, we investigated whether MAPK signaling pathways were necessary for Sema6A-induced neurite outgrowth. The p42/44 MAPK inhibitor U0126, p38 MAPK inhibitor SB203580, and c-jun NH_2_-terminal kinase (JNK) MAPK inhibitor SP600125 each inhibited NGF or Sema6A induced neurite outgrowth, as well as the synergistic effect of NGF/ Sema6A (Figure 2A–C). This suggested that MAPK pathway activation was critical to the neurotrophic action of Sema6A. We further investigated MAPK phosphorylation in PC12 cells stimulated by NGF, Sema6A, or NGF/Sema6A (Figure 2E). Either NGF or Sema6A dramatically increased p42/44 MAPK activation in PC12 cells within 5 min, with peak levels after 15 min and lasting for at least 60 min. NGF given together with Sema6A induced greater p42/44 MAPK activation. Similarly, both NGF and Sema6A stimulated a modest JNK MAPK activation, beginning within 5 min and lasting for at least 60 min. These results suggested that, in PC12 cells, Sema6A-stimulated NGF-induced neurite outgrowth was mediated by MAPK signaling pathways.

Most existing understanding of semaphorin signal transduction, in cooperation with plexin receptors, originated from researches of Semaphorin 3A and Semaphorin 4D in neuronal cells [31,32]. Semaphorin signaling results in growth cone repulsion during nervous system development because of activation of R-Ras GAP activity of the intracellular domains of plexinA1 and plexinB1. This, in turn, can inhibit integrin function by suppressing PI3K signaling [31,32]. Such evidence indicated that sustained PI3K activation could induce neurite outgrowth in PC12 cells and that inhibition of PI3K activity would inhibits neurite differentiation, before their formation [33,34]. Thus, we investigated whether the PI3K signaling pathway was involved in Sema6A-stimulated NGF-induced neurite outgrowth in PC12 cells. The PI3K inhibitor LY294002 substantially inhibited neurite outgrowth induced by NGF, Sema6A, or NGF/Sema6A in PC12 cells (Figure 2D), suggesting that like the MAPK pathway, activation of PI3K was also critical to the neurotrophic effects of Sema6A.

### 2.3. Rif Expression Antagonized Neurotrophin-Induced Neurite Outgrowth in PC12 Cells

**Figure 3:**
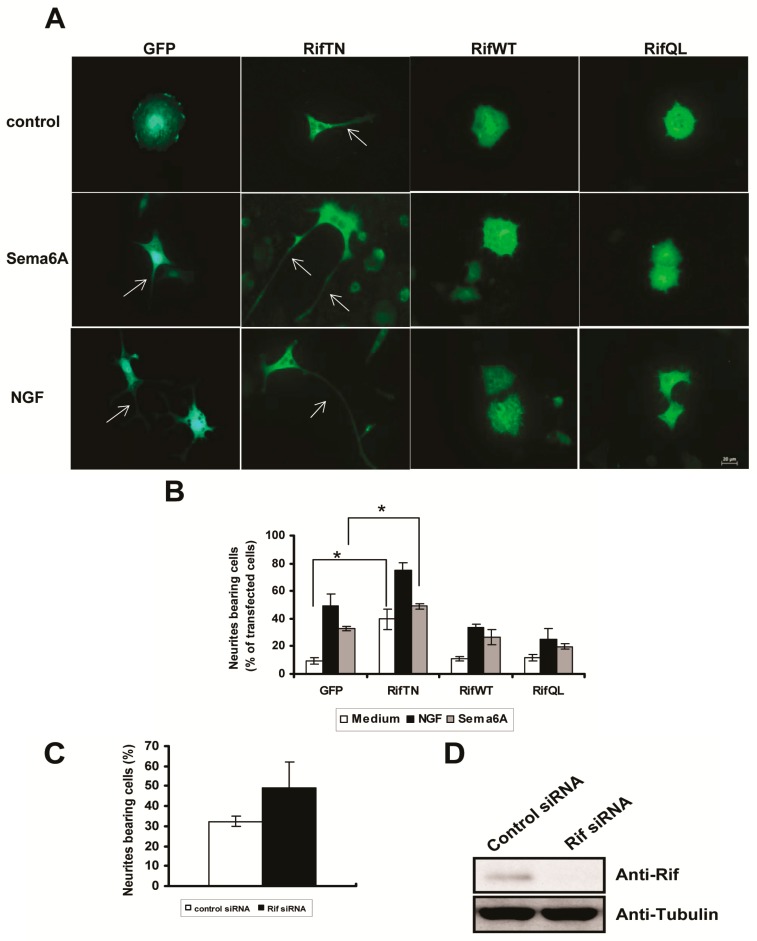
Rif expression antagonized neurite outgrowth induced by either NGF or Sema6A in PC12 cells. (**A**,**B**) Rif inhibited both NGF- and Sema6A-induced neurite outgrowth in PC12 cells. PC12 cells were transiently transfected with pEGFP-N3, the inactive Rif-TN mutant, wildtype Rif or the activated Rif-QL mutant using Lipofectamine 2000. At 24 h after transfection, cells were stimulated with either 50 ng/mL NGF or 1 nM Sema6A-Fc recombinant protein for another 24 h. At 48 h after transfection, cells were fixed and stained with an anti-Myc antibody. Cells were observed under an inverted microscope (Zeiss, Dresden, Germany). Arrows indicate the represented cells with neurites. Neurite outgrowth was quantified in positively-stained cells by counting those cells bearing at least one neurite with a length longer than twice the cell body diameter (Scale bar = 20 µm). Each value is the mean ± S.E. for >100 PC12 cells from three independent experiments. * *p* < 0.05, by Student’s *t* test; (**C**,**D**) Silencing endogenous Rif also induced neurite outgrowth in PC12 cells. PC12 cells were transiently transfected with control siRNA or Rif siRNA using Lipofectamine 2000. At 24 h after transfection, neurite outgrowth was assessed as described for (**A**,**B**). Endogenous expression of Rif and tubulin after siRNA transfection was determined by western blotting.

Neurite formation in PC12 cells involves cytoskeletal rearrangements. Small Rho GTPases, including RhoA, Rac, Cdc42, RhoG, and the Rnd subfamily members, have regulatory, and sometimes contradictory, effects in this process [12,13,14,35]. We found that stimulation of PC12 cells with Sema6A or NGF caused robust neurite outgrowth. Furthermore, expression of either wildtype Rif or the activated Rif-QL mutant inhibited neurite formation in response to Sema6A or NGF (Figure 3A,B). In contrast, transient expression of the inactive Rif-TN mutant was sufficient to significantly induce neurite outgrowth in the absence of Sema6A or NGF (Figure 3A,B), probably by competing with endogenous Rif. Not surprisingly, neurite outgrowth in PC12 cells was also induced by using siRNA to silence endogenous Rif (Figure 3C,D). We concluded that Rif antagonized neurotrophin-induced neurite outgrowth in PC12 cells.

### 2.4. Rif Activity Inhibited the Mitogenic Stimulation Mediated by Mitogen-Activated Protein Kinases (MAPKs) and Phosphatidylinositol-3-Kinase (PI3K) Activation

**Figure 4:**
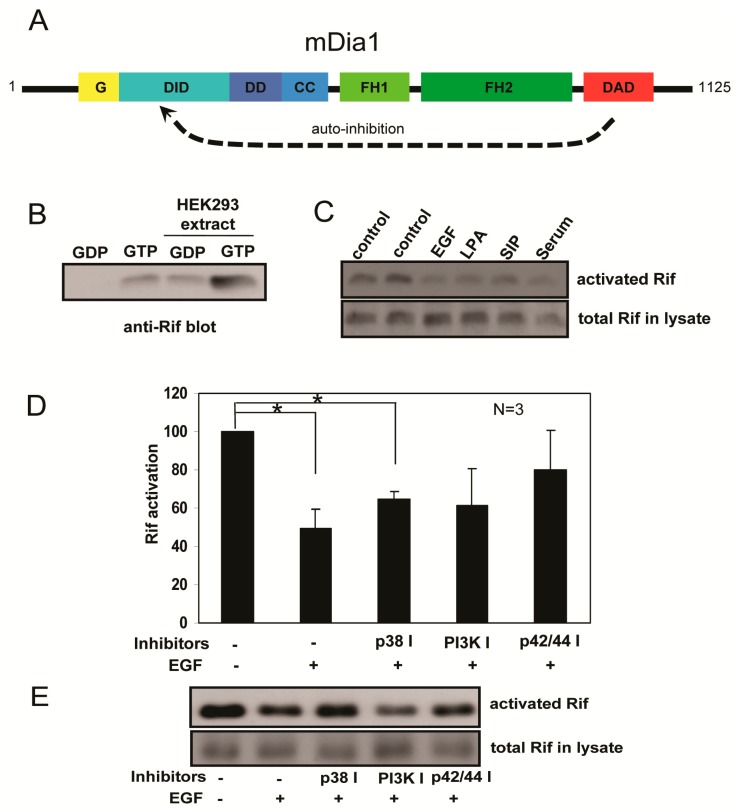
Rif activity was inhibited upon mitogenic stimulation mediated by MAPK or PI3K activation. (**A**) Domain structure of mDia1. Abbreviations: G, GTPase binding region necessary for RhoA binding; DID, diaphanous inhibitory domain; DD, dimerization domain; CC, coiled coil; FH1, formin homology 1 domain; FH2, formin homology 2 domain; DAD, diaphanous autoinhibitory domain; (**B**) pGEX-4T1-RifWT (1–195 aa) and pGEX-4T1-mDia1-G-DID (73–370 aa) recombinant proteins were generated in an *E. coli* protein expression system and soluble protein was immobilized to glutathione-Sepharose 4B beads for the use in a Rif activation assay. GST-Rif (1–195 aa) was cleaved by thrombin and then the released Rif was loaded with GDP or GTP. mDia1-G-DID beads were then incubated with GDP- or GTP-loaded Rif at 4 °C for 1 h. The beads were washed three times with lysis buffer and eluted with sample buffer for immunoblotting. HEK293 cell lysate was added to mimic the complex intracellular environment; (**C**) HeLa cells were serum starved overnight, then treated with 100 ng/mL Epidermal Growth Factor (EGF), 8 ng/mL Lysophosphatidic Acid (LPA), 1 µg/mL Sphingosine 1-Phosphate (SIP) or 10% serum for 3 min and Rif activation levels then examined by the Rif activation assay described in (**B**); (**D**) HeLa cells were serum starved overnight, then pretreated with or without p38 MAPK, PI3K, or p42/44 MAPK inhibitors for 30 min and then stimulated with 100 ng/mL EGF for 5 min. Rif activation levels were examined by the Rif activation assay. Rif activation levels were quantified by immunoblotting. Data are means ± S.E.M (*n* = 3). * *p* < 0.05, by Student’s *t* test; (**E**) Representative western blot showing Rif activation under EGF stimulation, with or without inhibitors. Activated Rif protein levels are shown in the top panel and total Rif protein in the lysate in the bottom panel.

The Rho GTPases in their GTP-bound states can bind downstream effectors and trigger relevant signaling pathways. Activation of Rho GTPases can be quantified using Rho GTPase activation assays. In such assays, an interactive protein that binds specifically to the activated form of the target Rho GTPase is used to capture it onto a bead support. We established an effective Rif activation assay using the Rif-specific effector mDia1. mDia1 is composed of several functional domains, including: the N-terminal G region for GTPase binding (residues 73–131); DID domain for DAD autoinhibition (residues 131–377); DD domain for mDia1 dimerization (residues 377–452); a coiled-coil domain that follows DD; central FH1 and FH2 domains; and C-terminal DAD domain (Figure 4A). Autoinhibition of mDia1 can be caused by blocking its Rho GTPase binding site via the DAD domain. The G-DID region (residues 73–370) was identified as the minimum region required for RhoA binding [36]. We thus speculated that the G-DID region might be used to develop a Rif activation assay. GST-mDia1-G-DID recombinant protein was immobilized to glutathione-Sepharose beads and GST-Rif recombinant protein was cleaved by thrombin to separate GST and Rif. This recycled Rif protein was allowed to incorporate, by loading, either GDP or GTP. Next, the GST-mDia1-G-DID beads were incubated with Rif-GDP or Rif-GTP, and the bound protein was eluted and analyzed by western blotting with an anti-human Rif antibody. GTP-loaded Rif had greater affinity for mDia1-G-DID than GDP-loaded Rif (Figure 4B). HEK293 cell lysate was added to mimic the complex intracellular environment, with results showing that the assay would work properly under various conditions, including in cell lysates (Figure 4B).

Mitogenic stimuli such as EGF, LPA, and serum can trigger classical Rho GTPase activity, resulting in GTP loading and activation of downstream effectors [37,38,39]. Results of the Rif activation assays showed that Rif activation was primarily decreased in mitogen stimulated HeLa cells (Figure 4C). Therefore, we further investigated Rif activation in PC12 cells stimulated with NGF or Sema6A. However, because of low endogenous Rif expression in PC12 cells, we detected no significant difference in Rif activation. However, it was reported that EGF and NGF adopt the same signaling pathway to mediate PC12 proliferation and differentiation, respectively [29], so we used EGF-stimulated HeLa cells to test Rif activation. We found that EGF treatment for 5 min led to an approximate 50% decrease in Rif activation. In cells treated with inhibitors of p38 MAPK, PI3K, or p42/44 MAPK Rif activation levels could be reversed to a different extent relative to the EGF treatment group (Figure 4D,E). We thus speculated that Sema6A-stimulated NGF-induced neurite outgrowth in PC12 cells was partially mediated by inhibition of Rif GTPase activity, downstream of MAPK and PI3K activation.

### 2.5. The Roles of Rif in PC12 Cell Neurite Formation

**Figure 5:**
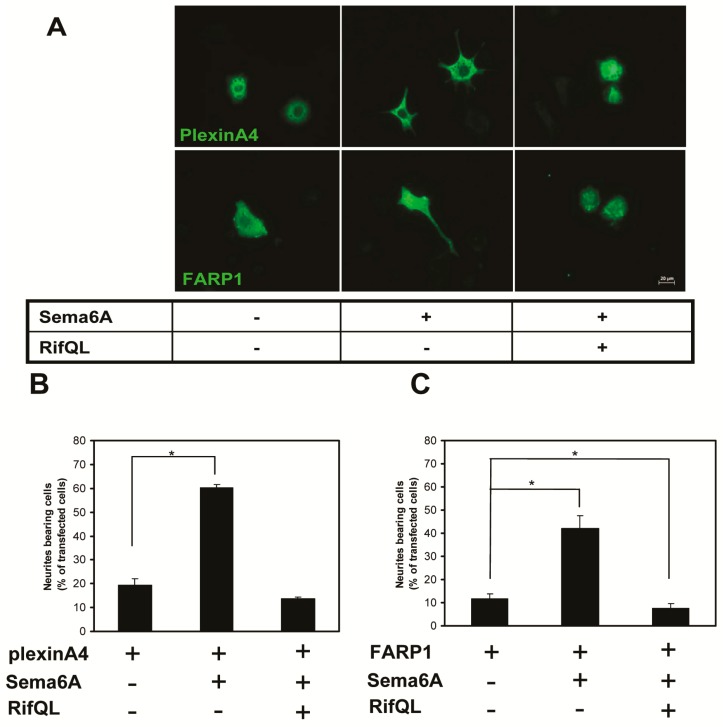
Rif antagonized Sema6A-induced neurite outgrowth in cells overexpressing plexinA4 or FARP1. (**A**) PC 12 cells were transiently double-transfected with plexinA4/RifQL (**top** panel) or FARP1/RifQL (**lower** panel) using Lipofectamine 2000. At 24 h after transfection, cells were stimulated with 1 nM Sema6A-Fc recombinant protein for another 24 h. Cells were then fixed, stained with the indicated antibodies, and observed under an inverted microscope (Zeiss); (**B**,**C**) Neurite outgrowth assessed in double-stained cells. Neurite outgrowth was quantified by counting PC12 cells bearing at least one neurite with a length longer than twice the cell body diameter (Scale bar = 20 µm). Each value is the mean ± S.E. for >100 PC12 cells from three independent experiments. * *p* < 0.05, by Student’s *t* test.

We previously showed that Rif GTPase regulated cytoskeletal signaling from plexinA4 to promote neurite retraction by controlling association between plexinA4 and FARP1. We also showed that regulation of FARP1 by Rif promoted neurite retraction in PC12 cells stimulated with Sema6A [26]. In the present study, we further investigated whether Rif would strongly antagonize Sema6A-induced neurite outgrowth, even with plexinA4 or FARP1 overexpressed. We transfected PC12 cells with plexinA4 or FARP1, together with RifQL. We then counted cells with neurites that were also double-transfected with plexinA4/RifQL or FARP1/RifQL. Even in the presence of plexinA4 or FARP1 overexpression, Rif still significantly inhibited neurite outgrowth in response to Sema6A (Figure 5). This indicated that the cells were very sensitive to levels of Rif activation. Therefore, Rif activation might represent a mechanism for fine-tuning neurotrophin-induced neurite formation in PC12 cells.

## 3. Discussion

Our study showed that PC12 cells were useful as a model system to dissect the pathways that mediate stimulation of neurite outgrowth by Sema6A and its inhibition by Rif GTPase. Sema6A is as potent as NGF for inducing neurite outgrowth in PC12 cells. Moreover, MAPK and PI3K signaling pathways are shared by Sema6A and NGF too, and can decrease Rif activation in cells. We thus concluded that Sema6A-stimulated NGF-induced neurite outgrowth in PC12 cells could be partially mediated by inhibition of Rif GTPase activity, downstream of MAPK and PI3K activation.

The emergence of non-classical members of the Rho GTPase family, such as Rif, has reflected development of vertebrates, including their increasing complex nervous system, adaptive immune system, and many other processes requiring regulation of the cytoskeleton and cell migration [21]. Rif has a broad tissue distribution pattern and Rif mRNA expression was observed in various human tissues of the nervous system, including fetal whole brain, adult whole brain, cerebral cortex, frontal lobe, hippocampus, and spinal cord [22]. As direct evidence of the importance of Rif in nervous system development, it mediated dendritic spine neck formation through its effector, mDia2, in hippocampal neurons [23]. Effects of semaphorins on the cytoskeleton are mediated in part by small GTPases [31,40,41], with balances in Rho and Rac activities determining growth cones collapse, dendrite formation, or neurite extension and retraction [42,43]. In our study, we newly identified Rif as a regulator of cytoskeletal rearrangements downstream of semaphorin signaling.

**Figure 6:**
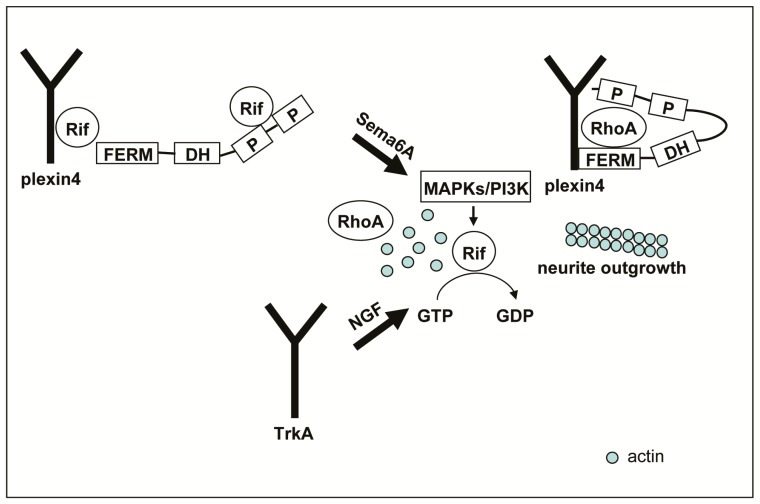
The role of Rif in neurotrophin-induced neurite formation in PC12 cells. In the absence of neurotrophin, the cells are maintained in a steady state and Rif protein exists in its highly activated form in the plasma membrane. When cells are stimulated with neurotrophin, MAPK and PI3K signaling pathways are activated, leading to decreased Rif activation. Sema6A and NGF activated signaling pathways could work individually or cooperatively by regulating downstream MAPKs/PI3K, to stimulate neurite outgrowth in PC12 cells.

Most Rho small GTPases cycle between GTP-bound active and GDP-bound inactive forms, to regulate the actin cytoskeleton, cell migration, cell motility, cell polarity, gene expression, vesicle trafficking, cell cycle regulation, and nervous system development [44]. The intrinsic GTP hydrolysis rate was shown to be much faster than intrinsic nucleotide dissociation, indicating that most GTPases exist in their inactive GDP-bound forms in the steady state [45]. However, in a study aimed to decipher the molecular and functional basis of Dbl family proteins, it was demonstrated that the Dbl proteins investigated showed no Guanine-nucleotide Exchange Factor (GEF) activity for Rif and RhoD [46]. It was speculated that, unlike the conventional Rho GTPases, Rif and RhoD might persist in the GTP-bound state under resting conditions. Our measurements of Rif activation levels in cells under exogenous mitogen stimulation indicated that Rif-GTP levels were higher before, than after, EGF stimulation (Figure 4). In addition, endogenous Rif could not be loaded with GTPγS, because a high percentage was already bound to GTP in the steady-state (Figure S1). We thus proposed a working model for involvement of Rif in neurite formation in PC12 cells (Figure 6). In the absence of a neurotrophin (such as Sema6A), cells are maintained under a steady state and Rif protein remains highly activated form in the plasma membrane. This leads to disassociation of plexinA4 and FARP1, releasing trapped active RhoA to the cytoplasm. Under these conditions, neurite retraction can be induced. When cells are stimulated with a neurotrophin, such as semaphorins or NGF, MAPK and PI3K signaling pathways are activated, leading to suppression of Rif activation. There is then insufficient activated Rif to interact with FARP1, resulting in re-association of plexinA4 and FARP1, trapping active RhoA in the plexinA4/FARP1 complex. This enables neurite outgrowth to be induced.

The distinguishing feature of Rif GTPase is that it might be highly activated under the steady state, unlike RhoA and Rac, which usually stay in their GDP bound forms in the absence of stimulation. Our findings demonstrated that Sema6A and NGF signaling pathways could work either individually or cooperatively to regulate downstream MAPKs/PI3K signaling, decrease Rif activation, and stimulate neurite outgrowth in PC12 cells. In addition to its well-known functions during neural development, NGF was also shown to have regenerative properties following trauma, through both downregulation of chemorepulsion and upregulation of outgrowth cues [47,48]. NGF was reported to have specific impact on the effects of Sema3A, whose repulsive effects were mediated by NGF concentration gradients [49,50]. Our results showed that Sema6A could also influence the effects of NGF by increasing sensitivity of PC12 cells to NGF. This raised the possibility that NGF and semaphorin family members could be combined, in the clinic, for treating of neurological diseases. It will be of interest to further investigate Rif GTPase as a new target for modulating neuronal differentiation and neurological disorders.

## 4. Materials and Methods

### 4.1. Materials

Monoclonal antibodies to myc epitope, p-ERK1/2 (12D4) and p-JNK (G-7) were from Santa Cruz Biotechnology (Santa Cruz, CA, USA). Mouse anti-tubulin antibody was from TransGen Biotechnology (Beijing, China). Polyclonal antibody to Rif was from Abcam (Cambridge, UK). Alexa Fluor^®^ 594 phalloidin and goat anti-mouse IgG (H + L) secondary antibody (Alexa Fluor^®^ 488 conjugate) were from ThermoFisher Scientific (Waltham, MA, USA). Recombinant human epidermal growth factor (Hu EGF) was from ThermoFisher Scientific. Nerve growth factor-7S (NGF) from mouse submaxillary glands was from MilliporeSigma (Darmstadt, Germany). Recombinant human semaphorin 6A Fc chimera was from R&D systems (Minneapolis, MN, USA). Glutathione-Sepharose 4B beads were from GE Healthcare (Fairfield, CT, USA). Plasmids encoding N-terminal epitope-tagged human RifWT, the inactive RifT33N mutant and the constitutively active RifQ77L mutant in pcDNA3 (Clontech, San Francisco, CA, USA) were described previously [21,22,51]. A full list of plasmids is shown in supplementary materials (Table S1). K-252a was from Biomol (Heidelberg, Germany). U0126 and LY294002 were from Cell Signaling (Minneapolis, MA, USA). SB203580 and SP 600125 were from MilliporeSigma (Darmstadt, Germany). All reagents were used at concentrations shown in the literature to substantially block their respective targets.

### 4.2. Cell Culture

HeLa cells were maintained in Dulbecco’s modified Eagle’s medium (DMEM) supplemented with 10% (*v*/*v*) heat-inactivated fetal bovine serum (FBS, Invitrogen, Waltham, MA, USA), 100 µg/mL streptomycin sulfate and 100 U/mL penicillin solution (Gibco, Waltham, MA, USA). PC12 cells were maintained in Roswell Park Memorial Institute Medium (RPMI)-1640 containing 5% FBS, 5% horse serum (Gibco), 100 µg/mL streptomycin sulfate, and 100 U/mL penicillin solution. Cells were seeded into 10 cm^2^ tissue culture plates (Corning, Corning, NY, USA) and cultivated at 37 °C in 95% air, 5% CO_2_, and at 90% humidity.

### 4.3. Determination of Neurite Outgrowth

In neurite outgrowth experiments with stimulation by Sema6A and/or NGF, 4 × 10^4^ cells were added to each well of a 12-well plate previously coated with poly-l-lysine. After 24 h, medium was replaced with fresh serum-free medium and cells stimulated with 50 ng/mL NGF and/or 1 nm Sema6A for 24 h. The cytoskeleton was then stained with Alexa Fluor^®^ 594 phalloidin and cells fixed for 15 min in 4% paraformaldehyde and processed for immunofluorescence.

In neurite outgrowth experiments with various inhibitors, 4 × 10^4^ cells were added to each well of a 12-well plate previously coated with poly-l-lysine. After 24 h, medium was replaced with fresh serum-free medium and cells pretreated with K-252a (100 nm), U0126 (10 µm), SB20358 (10 µm), SP 600125 (50 µm), or LY294002 (50 µm) for 30 min. Next, 50 ng/mL NGF and/or 1 nm Sema6A were added to the medium and cells incubated for another 24 h. The cytoskeleton was then stained with Alexa Fluor^®^ 594 phalloidin, then cells were fixed for 15 min in 4% paraformaldehyde and processed for immunofluorescence.

In neurite outgrowth experiments with Rif protein expression, 4 × 10^4^ cells were added to each well of a 12-well plate previously coated with poly-l-lysine. The next day, PC12 cells were transfected with different Rif constructs, using Lipofectamine 2000 (ThermoFisher). After 24 h, 50 ng/mL NGF and/or 1 nm Sema6A were added to the medium and cells incubated for another 24 h. Cells were then fixed for 15 min in 4% paraformaldehyde and processed for immunofluorescence.

Cells extending at least one neurite with a length longer than twice the cell body diameter were counted as neurite-bearing cells, as previously described [52]. For quantification of neurites, >100 cells were analyzed in each experiment and each experiment was repeated three times. Statistical significance was analyzed using Student’s *t* test (two-tailed).

### 4.4. siRNA Transfection

The siRNA oligonucleotides were transfected into PC12 cells using calcium phosphate. In brief, cells were cultured in 12-well or 6-well plates until 70% confluent. For 6-well plates, as an example, transfection solutions were composed of mixing 4 µL 20 µm siRNA in 100 µL 0.25 M CaCl_2_ with 100 µL 50 mm *N*,*N*-bis(2-hydroxyethyl)-2-aminoethanesulfonic acid (BES), pH 6.9, 280 mm NaCl and 1.5 mm Na_2_HPO_4_. After incubation for 15 min, this suspension was added dropwise to each well of the culture dish. Samples were incubated at 37 °C in 97% air, 3% CO_2_ overnight. The medium was then replaced twice and samples incubated at 37 °C in 95% air, 5% CO_2_ for a further 48 h. The sequences of siRNA oligonucleotides were: control, 5′-UUCUCCGAACGUGUCACGUTT-3′; Rif, 5′-CCUGAAGUCACGCAUUUCUTT-3′.

### 4.5. Preparation of Recombinant Protein and Rif Activation Assay

mDia1 is an actin nucleator protein that nucleates actin filament polymerization at the barbed ends. RhoA binding to the RBD of mDia1 relieves the autoinhibition between the DAD and GBD-DID domains, allowing actin nucleation [53]. Purified mDia1-G-DID had a higher affinity for Rif-GTP than Rif-GDP, so we selected mDia1-G-DID as the bait for activated Rif protein.

*Escherichia coli* was transformed with GST-tagged mDia1-G-DID. Cells were induced with 0.1 mm isopropyl β-d-1-thiogalactopyranoside (IPTG) for 4 h. They were then lysed in ice-cold extraction buffer (50 mm Tris, pH 7.5, 50 mm NaCl, 5 mm MgCl_2_, 1 mm GDP and 1 mm DTT) by nebulization. The lysate was clarified by centrifugation at 17,400× *g* for 30 min and loaded onto glutathione-coupled Sepharose beads. GST-Rif (1–195 aa) beads were washed three times with thrombin cleavage buffer (1 µm GDP, 50 mm Tris pH 7.5, 50 mm NaCl, 5 mm MgCl_2_ and 1 mm DTT). Thrombin (2 active unit per 100 µL protein) was added and samples incubated at 4 °C overnight with gentle rotation. The next morning, 30 µL glutathione-Sepharose beads and 30 µL *p*-aminobenzamidine was added to the cleavage reaction supernatant to remove remaining GST and thrombin, respectively. Cleaved Rif protein was quantified by the BCA assay, according to the manufacturer’s protocol (Life Technologies, Carlsbad, CA, USA). Purified Rif protein was diluted in the exchange buffer (20 mm Hepes pH 7.5, 10 mm EDTA, 100 mm NaCl, and 1 mm DTT) to deliver 0.1 µg Rif for each exchange assay. Then, 6 µL 100 mm GDP (1 mm final concentration) or 6 µL 10 mm GTP (100 µm final concentration) was added to the beads and gently mixed by pipetting. The beads were incubated at 4 °C for 30 min with gentle shaking. The exchange process was terminated by adding 40 µL 1 M MgCl_2_ to each tube (>60 mm final concentration). mDia1-G-DID beads were sedimented by brief centrifugation and supernatants discarded. Beads were then incubated with GDP-or GTP-loaded Rif at 4 °C for 1 h. The beads were washed three times using lysis buffer and eluted with sample buffer for immunoblotting. Extra HEK293 cell lysate was added to mimic the complex cellular environment. Rif activation assays were performed as described previously for RhoA activation assays [37].

## 5. Conclusions

In this study, we demonstrated that Sema6A was as effective as NGF for stimulating neurite outgrowth in PC12 cells and that its neurotrophic effects were transmitted by MAPK and PI3K signaling. We further showed that neurotrophin-induced neurite formation in PC12 cells might be partially mediated by inhibition of Rif GTPase activity, downstream of MAPK and PI3K signaling. We newly identified Rif as a regulator of cytoskeletal rearrangements mediated by semaphorins.

## Supplementary Materials

Supplementary materials can be found at www.mdpi.com/1422-0067/18/1/148/s1.
