# Supplementary material for: Neurotrophin Promotes Neurite Outgrowth by Inhibiting Rif GTPase Activation Downstream of MAPKs and PI3K Signaling
Source: Int J Mol Sci. 2017 Jan 13;18(1):148. doi: 10.3390/ijms18010148 (PMC5297781; doi:10.3390/ijms18010148)
Supplement: Supplementary file 1 [file ijms-18-00148-s001.pdf]

Int. J. Mol. Sci. **2017**, *18*, 148; doi:10.3390/ijms18010148

Supplementary Materials: Neurotrophin Promotes Neurite Outgrowth by Inhibiting Rif GTPase Activation Downstream of MAPKs and PI3K Signaling

Xiaoxia Tian, Huijuan Yan, Jiayi Li, Shuang Wu, Junyu Wang and Lifei Fan

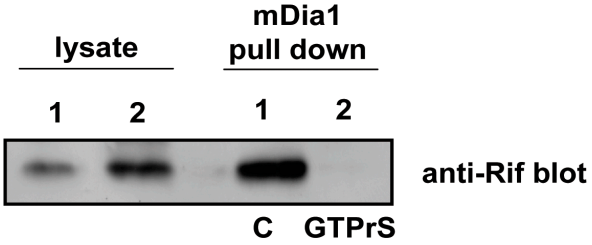

Figure S1. Rif might persist in the guanosine 5′-triphosphate (GTP)-bound state under resting situation.

HeLa cells were serum starved overnight, and then two 10 cm^2 of cells were lysed with the lysis buffer D (50 mM Tris.Cl pH 7.2, 1% Triton X-100, 500 mM NaCl, 0.1 mM PMSF, ethylene diamine tetraacetic acid (EDTA) free protease inhibitors cocktail). The first lysate (labeled as 1) were left untreated and the second lysate (labeled as 2) were loaded with GTPγS. To load GTPγS, 6 μL of 10 mM GTPγS (≥100 μM final concentration), 12 μL of 0.5 M EDTA pH 8.0 (≥10 mM final concentration) were added to the supernatant. Rotate 30 min at 4 °C. The exchange reaction was terminated by adding 38 μL of 1 M $MgCl_2$ to each tube. 200 μL miDia1-G-DID beads were added to each of the tubes and rotate 45 min at 4 °C. The beads were washed three times with wash buffer and followed by immune blotting analysis. The endogenous Rif could not be loaded with GTPγS due to its high GTP binding percentage in the steady status.

Table S1. Plasmid constructs used in the study.

Construct	Residues	Mutations	Vector	Source
pEGFP-N3	Full length	-	pEGFP-N3	Lab storage
myc-Rif WT	Full length	-	pcDNA3	[21]
myc-Rif QL	Full length	Glutanine(Q)77 > Leucine(L)	pcDNA3	[21]
myc-Rif TN	Full length	Threonine(T)33 > Asparagine(N)	pcDNA3	[21]
myc-plexinA4	Full length	-	pCAG	Sockanathan
GST-mDia1-G-DID	73–370 aa	-	pGEX-4T1	Rosen
pGEX-4T1-RifWT	1–195 aa	-	pGEX-4T1	Ahmadian
HA-FARP1	Full length	-	pcDNA3	[26]
